# Microbial spectrum and drug resistance profile in solid malignancies in a large tertiary hospital from Palestine

**DOI:** 10.1186/s12879-022-07375-6

**Published:** 2022-04-18

**Authors:** Rama Rabayah, Ranyah B. Alsayed, Adham Abu Taha, Husam Salameh, Riad Amer, Ali Sabateen, Banan M. Aiesh, Sa’ed H. Zyoud

**Affiliations:** 1 Department of Medicine, College of Medicine and Health Sciences, An-Najah National University, Nablus, 44839 Palestine; 2 Department of Biomedical Sciences, Faculty of Medicine and Health Sciences, An-Najah National University, Nablus, 44839 Palestine; 3 Department of Pathology, An-Najah National University Hospital, Nablus, 44839 Palestine; 4 Department of Hematology and Oncology, An-Najah National University Hospital, Nablus, 44839 Palestine; 5 Infection Control Department, An-Najah National University Hospital, Nablus, 44839 Palestine; 6 Department of Clinical and Community Pharmacy, College of Medicine and Health Sciences, An-Najah National University, Nablus, 44839 Palestine; 7 Poison Control and Drug Information Center (PCDIC), College of Medicine and Health Sciences, An-Najah National University, Nablus, 44839 Palestine; 8 Clinical Research Center, An-Najah National University Hospital, Nablus, 44839 Palestine

**Keywords:** Antimicrobial resistance, Solid tumors, Infection

## Abstract

**Background:**

Since the available data for bloodstream infections in solid malignancy tumors are somewhat limited in Palestine, prevention of infection before the occurrence, controlling it when it occurs, and implementing stewardship programs are important ways in the whole therapy of solid tumor patients, which is becoming challenging recently with the evolution of more antimicrobial drug-resistant pathogens. Therefore, our study aims to assess the microbial spectrum and antimicrobial sensitivity and the overall outcome related to many clinical risk factors in patients with solid tumor patients seeking care in a referral hospital as an experience from a developing country.

**Methods:**

From the onset of 2018 to the end of 2020, a total of 116 episodes with positive blood cultures were retrospectively studied and analyzed in 96 patients who had solid tumors in a referral hospital in Palestine.

**Results:**

We identified 116 positive blood cultures in 96 patients with a male to female ratio of 1:1. The mean age was 58 years. Breast cancer was the tumor most frequently recorded (13.5%), followed by urinary tract tumors (10.4%). The most common source of episodes with positive blood culture was catheter-related. Gram-positive bacteria accounted for 52.6% of blood cultures with the predomination of Staphylococcus species. On the contrary, Gram-negative bacteria were documented in 39.7% of the cultures, with *E. coli* being the most frequent bacteria. Regarding fungi that were only *Candida species*, it was isolated in 15.5% of the cultures.28.4% of patients started on a single antimicrobial as an initial regimen, the remaining started combination antimicrobial therapy. The initial antimicrobials used most frequently were aminoglycosides in 29.3% of the episodes. All species of *Staphylococcus* were sensitive to vancomycin. *Enterococcus species* were fully resistant to ciprofloxacin. In the case of *E. coli,* the isolates were 100% sensitive to imipenem, meropenem, and amikacin and were mostly resistant to ampicillin, where the sensitivity was only about 19.5%. *P.aeruginosa* was sensitive in 83.3% of cultures to both piperacillin-tazobactam and gentamicin, but highly resistant to imipenem, in which sensitivity decreased to 50%. The isolates of *Klebsiella species* were 72.2% sensitive to gentamicin, meropenem, and imipenem and 100% resistant to ampicillin. *A. baumannii* was 50% sensitive to trimethoprim-sulfamethoxazole. Candida species showed high sensitivity to both caspofungin and flucytosine (83.3%), followed by 77.8% sensitivity to voriconazole. Death was reported in 27.6% of the episodes and there was a significant relationship between shock at presentation and death (*p* = 0.010).

**Conclusions:**

The findings of this investigation confirm the prevalent BSI seen in patients with solid malignancies and demonstrate a significant percentage of antibiotic resistance. Therefore, stewardship programs that dig deep before using any type of antimicrobials will help reduce the risk of resistance to antibiotics. In addition, the implementation of infection control surveillance plays an important role in decreasing the risk of contamination.

**Supplementary Information:**

The online version contains supplementary material available at 10.1186/s12879-022-07375-6.

## Background

Solid malignancy patients are at high risk of catching microbes due to the hospital's long stays fluctuations in their immune system due to invasive diagnostic and therapeutic procedures they undergo [1]. Therefore, bloodstream infections (BSIs), which are defined as the presence of bacterial, fungal, or viral pathogens in at least one blood culture, have been commonly identified in either patients with solid or hematologic malignancies. However, there was an obvious gap in the literature about solid malignancy patients compared to hematologic malignancy or bone marrow transplant recipients [2].

The microbial profiles and many prognostic factors play an important role in the overall cases fatality and management plan of these cases according to the type of regimen to the specific dose and the appropriate duration. Suppose that these risk factors are not taken seriously or missed in oncology patients. In that case, many consequences will be faced, including complex treatment regimens, prolonged hospital stays, a heavy financial burden on the health system, and increased morbidity and mortality rates [3]. Solid malignant patients have a high mortality rate, particularly in patients with other comorbidities, advanced stage tumors, a history of receiving chemotherapy or radiation therapy, a history of recent medications that affect the immune system and present with shock [4].

The mortality rate due to BSIs and their complications in patients is approximately 50% of deaths, but the administration aims to reduce the risk to approximately 10% [3]. Today, antibiotic resistance is of great importance due to its irrational use, which can be explained by easy and uncontrolled access, particularly in developing countries [5]. This continuous and unrestricted use contributes to the development of very resistant microorganisms known as multiple drug-resistant pathogens [5]. The precise definition of multiple drug-resistant pathogens is when it is resistant to at least one antibiotic from at least three classes of antibiotics [6]. In addition to antibiotic resistance, those high virulence organisms became one of the challenging issues in the treatment of cancer patients [3]. Antimicrobial stewardship programs aim to improve patient outcomes and reduce costs through the appropriate use of antibiotics [7]. A study conducted in Palestine showed a 24.3% decrease in antimicrobial use after the antimicrobial stewardship program [8].

According to previous studies, patients diagnosed with solid malignancy have shown antibiotic resistance and multiple episodes of infections with various microbial species that will affect their life expectancy, determining those in addition to the outcome of BSIs will give better vision in treating patients from the beginning of the course, which will decrease the length of stay in the hospital and as a result decrease the risk of catching nosocomial infections and will be cost-effective for the hospital itself. Today, there is a great improvement in the methods used to treat cancer patients. However, still, many patients die from infections rather than the tumor itself, so it is worth digging deep behind the source of the infection to decrease mortality and morbidity among these patients.

Patients diagnosed with malignancies are at increased risk for BSIs, leading to increased rates of morbidity and mortality among cancer patients and negatively impacting the dose and duration requested for antineoplastic chemotherapy [9]. In addition to that, patients are at increased risk of developing these infections, especially during chemotherapy treatment with chemotherapy [3]. Whatever the cause of the infection, this may contribute to the development of antimicrobial resistance and the treatment of high virulence organisms. Although there is a large improvement in survival in cancer patients, infections are still a major cause of morbidity and mortality. In addition, hospitals still deal with many cases of sepsis in the oncology department. If this issue is not taken seriously, many patients will enter the hospital for long periods, increasing the susceptibility to catch hospital-acquired microbes. Others will develop resistance to different antibiotics, including broad-spectrum antibiotics. The health system will face a financial burden for diagnostic studies, imaging, and treatment procedures from an economic point of view.

This study aims to evaluate the prevalence of BSIs in cancer patients so that it will focus on the microbial spectrum, antimicrobial sensitivity profile, predisposing and prognostic factors, clinical characteristics, microbiological characteristics, source and outcome of BSIs exclusively in patients with solid tumors treated in a referral hospital as an experience from a developing country.

## Methods

### Study design

A retrospective analysis study has been conducted in a hospital. We analyzed data collected from patient records diagnosed with solid malignancies and admitted to An-Najah National University Hospital (NNUH) in Palestine from the beginning of 2018 to the end of 2020. This study design was chosen because no intervention was needed; only the microbial spectrum, the antimicrobial resistance pattern, and the associated factors were required. Furthermore, there was no follow-up for a specific exposure, so no cohort study or case–control study was necessary.

### Ethical consideration

All aspects of the study protocol, including access to and use of patient clinical information, had been approved by the *Institutional Review Boards (IRB) of An-Najah National University*. Therefore, we confirm that the collected data were used only for clinical research. The information was confidential and was not used for any purpose other than this study. The data collected were only with limited access to the working staff in the project. Identifiable information of the patients was not shared; we used numbers as codes for patients instead of their names.

### Study population

Solid malignancy patients who treated in the oncology department of the An-Najah National University Hospital were our target population. Inclusion criteria: (1) solid malignancy patients at or above the age of 18 years old; and (2) developing BSI or sepsis after the diagnosis of malignancy has occurred. Exclusion criteria: (1) patients diagnosed with lymphoma; and (2) patients diagnosed with hematologic malignancies or skin cancer.(skin cancer is not followed up in the study hospital).

### Setting and study time

The study was carried out at An-Najah National University Hospital because it is one of the largest hospitals in Palestine and is considered the referral hospital for cancer cases in the country. The time spent for data collection and analysis was from the 1st of October 2020 to the 15th of March 2021.

### Sample size

This study took all solid malignancy patients admitted to the hospital and showed a positive blood culture during their stay between January 2018 and the end of 2020. Twenty patients were excluded from the study due to missing records or data. Therefore, data collected for 96 patients with 116 episodes with positive blood cultures were studied and analyzed.

### Laboratory methods

Daily CBC was taken from each patient; two peripheral blood cultures were taken if the patient showed clinical or laboratory suspicion of bloodstream infection. If the patients have a central line, one is peripheral, and the other is central. The CDC/NHSN surveillance definition of healthcare-associated infection and criteria for specific types of infection were used to determine infection classification.

### Microorganism Identification and Drug-Susceptibility Test

Blood specimens were inoculated into a VersaTrek Redox 1 aerobic and Redox 2 anaerobic media (Thermo Fisher Scientific, Waltham, MA) and analyzed by the automated microbial detection system (VersaTREK™, TREK Diagnostic Systems, Cleveland, OH, USA). Incubation was continued until a positive culture was observed or up to a maximum of 5 days. Positive bacterial cultures were tested for the type of pathogen and sensitivity to antibiotics. The identification of bacteria and antibiotic sensitivity was performed using VITEK 2® COMPACT (bioMérieux, Marcy-I'Étoile, France). Gram-negative and Gram-positive bacteria were identified using GN and GP cards, respectively. Antibiotic sensitivity of Gram-negative bacteria was performed using AST-GN204 and AST-GN222 labels. The sensitivity of Gram-positive was determined using AST-GP67 and AST-GP 03 cards. While Candida species were identified using the *VITEK2 YST ID* card and their susceptibility was determined using the *VITEK2* fungal susceptibility card AST-YS-08. Multiresistance was determined phenotypically from the antibiotic susceptibility profile using the VITEK 2® COMPACT. Interpretations of drug susceptibility data were based on the Clinical Laboratory Standards Institute standard (CLSI, United States).

### Data collection instrument

The patient’s records were reviewed for each admission with a positive blood culture. Data were collected and filled in the data collection form (Additional file 1). The data collection form included the following sections:

- Section 1 collected the patient’s age, type of solid cancer, sex, and previous medications.
- Section 2 was used to detect the number of episodes of BSIs episodes concerning the underlying type of tumor, the specific isolated pathogens, and its source.
- Section 3 studied whether specific factors increased the risk of BSI in patients. Furthermore, this section included information on comorbidities like diabetes mellitus, hypertension, chronic kidney diseases, inflammatory bowel disease, cardiac disease, thyroid disorders, pulmonary diseases, and neurological disorders. Immunosuppressive medications suppress the immune system other than chemotherapy, such as steroids, methotrexate, and azathioprine.
- Section 4 was used to know the specific pathogens that caused BSIs, classify them as Gram positive or negative, and identify the percentage causing BSIs and death for each type. Multidrug-resistant bacteria definition: bacteria which showed resistance to at least two antibiotic categories
- Section 5 collected clinical factors associated with mortality from developing BSI, susceptibility and resistance to antibiotics. The case mortality rate was defined as death within the same admission for clinical suspicion of bloodstream infection, which occurred from the beginning of 2018 to the end of 2020.

### Statistical analysis

IBM-SPSS version 21 was used to analyze the data. Descriptive analysis was used to describe demographic characteristics, clinical characteristics of patients, causative microbial organisms, empirical antimicrobial therapy, and the sensitivity of the organisms to antimicrobials. Their frequencies and percentages represented them. Furthermore, the minimum, maximum and mean with standard deviation for age were calculated. Finally, we used either the chi-square test or Fisher's exact test to assess a relationship between some risk factors and death. Depending on the resultant *p-value,* values less than 0.05 were considered statistically significant.

## Results

### Demographic data of patients

The study population included all solid malignancy patients with positive blood cultures, which counted 96 patients out of 871 solid malignancy patients admitted to the hospital from the beginning of 2018 to the end of 2020 with a prevalence of 11%; approximately 20 patients were excluded from the study due to lack or missing data and clinical records. The demographic characteristics of the study included the gender and age categories of the patients. Half of them were males; the most frequent age category in which BSI occurred was 60–69 years, followed by 50–59 years, as seen in Table 1.

**Table 1:** Demographic characteristics of solid malignancy patients with BSI

Demographic features	Frequency	Percent (%)
Gender		
Male	48	50
Female	48	50
Total	96	100
Age categories (Year)		
19–29	3	3.1
30–39	4	4.2
40–49	20	20.8
50–59	21	21.9
60–69	30	31.3
70–79	14	14.6
80–89	2	2.1
90–99	2	2.1

### Clinical characteristics of patients

Regarding the clinical characteristics of the patients, the study found that the breast tumor was the most common tumor type in 13.5% of the patients, followed by urinary tract tumors (10.4%) and colorectal cancer (9.4%). Furthermore, approximately 56% of the patients had comorbidities and approximately 42% had metastatic disease. Approximately 65% of the patients had a history of receiving chemotherapy in the last month of BSI episodes, and about 10% of them received radiation therapy in the last month of BSI episodes. Regarding recent medications used within the last month of BSI episodes, approximately 15% of the patients had been treated with corticosteroid therapy, 5.2% had received immunosuppressive medications, and 7.8% reported using hormonal therapy.

Approximately 58% of the patients received antimicrobials within the last month, and about (15%) of the episodes showed neutropenic fever at BSI. Depending on the source of infection, the most common source was the infection of unknown origin in approximately 39%, approximately 36% were catheter-related infections, and 31.9% of the total number of patients had the central line at the time of BSI, followed by the respiratory tract, cholangitis, skin, and soft tissue origin with a percentage of 6% for each.

About 22% of the patients presented with septic shock and about 53% had undergone a previous invasive procedure within ten days of the BSI episode. Most of the patients (82.3%) experienced a single episode of BSI, 14.6% had two episodes of BSI, and 3.1% had multiple episodes of BSI. Table 2 shows the clinical characteristics in detail.

**Table 2:** Clinical characteristics along with the type of solid malignancy in patients

Characteristic	Frequency	Percent (%)
Underlying tumor type		
Breast	13	13.5
Urinary tract^1^	10	10.4
Colorectal	9	9.4
Gynecological^2^	8	8.3
Pancreatic	8	8.3
Hepatobiliary	7	7.3
Lung	7	7.3
Gastric	7	7.3
Sarcoma	6	6.3
Prostate	5	5.2
Small bowel	5	5.2
Others^3^	4	4.2
Esophageal	3	3.1
Head and neck^4^	3	3.1
Testicular	1	1
Metastatic disease	40	41.7
Comorbidities	54	56.3
Neutropenic fever	16	13.8
Previous chemotherapy (during 1 month)	75	64.7
Previous radiotherapy (during 1 month)	12	10.3
Previous invasive procedure (during 10 days)	62	53.4
Recent medications (1 month)		
Corticosteroid therapy	17	14.7
Immunosuppressive medications*	6	5.2
Hormonal therapy	9	7.8
Previous antimicrobial therapy	67	57.8
Source of BSI		
Unknown	45	38.8
Catheter related^5^	42	36.2
Skin and soft tissue	7	6
Respiratory tract infections	7	6
Cholangitis	7	6
Urinary tract	6	5.2
Other sites^6^	2	1.7
Presence of central line	37	31.9
NO. of BSI		
One episode	79	82.3
Two episodes	14	14.6
Multi-episodes	3	3.1

^1^Urinary tract: Renal, ureter and bladder cancer

^2^Gynecological tumors: include ovarian and uterine cancer

^3^Other types of cancer: mediastinal, brain, mesothelioma, and unknown origin

^4^Head and neck: Thyroid, thymus, and maxillary sinus cancer

^5^Catheter-related infection: central line,peripheral line, full catheter, port catheter, perm catheter

^6^Other sites: intra-abdominal infection and follicular tonsillitis

*Immunosuppressive medications other than chemotherapy, such as steroids, methotrexate, and azathioprine

Gram-positive organisms represented 52.6% of the cases. The most frequent Gram-positive bacteria were *Staphylococcus species* that represented 38% *(S. aureus* (4.3%)*, S. epidermidis* (14.7%) and other *Staphylococcus species* (19%), followed by *Enterococcus species* 6.9% and *Micrococcus luteus* 4.3%. Among gram-negative organisms (39.7%), *E. coli* were the most frequently isolated (18.1%), followed by *Klebsiella species* (15.5%) and *P. aeruginosa* (5.2%) and *A. baumannii* (5.2%). Fungal infection has been identified to occur in approximately 15.5% of BSI episodes, and *Candida species* was the only fungus identified from blood cultures. The results showed that of the five episodes of BSI with a positive culture for *S. aureus,* two were resistant to methicillin. This study found that multi-drug resistant bacteria, which means; that the bacteria show resistance to at least two antibiotic categories, accounted for about 15.5%. Of the 116 suspected BSI episodes, 13 cultures showed polymicrobial; this means that the same culture contains different organisms or the same bacteria of different species. More details can be found in Table 3.

**Table 3:** Detected organisms in each culture of suspected blood stream infection

Causative organisms	Frequency	Percent (%)
Gram-positive bacteria	61	52.6
*Staphylococcus aureus*	5	4.3
*Staphylococcus epidermidis*	17	14.7
*Other staphylococcal species*^*1*^	22	19
*Enterococcus species*^*2*^	8	6.9
*Actinomyces*	2	1.7
*Micrococcus luteus*	5	4.3
*Bacillus species*	1	0.9
*Other pathogens*	3	2.6
Gram negative bacteria	46	39.7
*Escherichia coli*	21	18.1
*Pseudomonas aeruginosa*	6	5.2
*Klebsiella species*^*3*^	18	15.5
*Acinetobacter baumannii*	6	5.2
Fungi	18	15.5
*Candida species*^*4*^	18	15.5
Multi drug resistant bacteria	18	15.5
Polymicrobial BSI^5^	13	11.2
Methicillin-resistant Staphylococcus aureus	2	40

^1^*Staphylococcus capitis, Staphylococcus haemolyticus, Staphylococcus hominis, Staphylococcus cohnii, Staphylococcus warneri*

^2^*Enterococcus faecalis, Enterococcus durans*

^3^*Klebsiella pneumonia, Klebsiella oxytoca*

^4^*Candida glabarta, Candida parapsilosis, Candida albicans, Candida krusei, Candida kefyr, Candida tropicalis, Candida dubliniensis*

^5^Polymicrobial: growth of two or more organisms in the same blood culture

All patients received empirical antimicrobials, which are defined as antimicrobials given to the patient before the culture results are ready. It was noticed, as seen in Table 4, that 71.6% were on a combination therapy regimen and 28.4% received a single antimicrobial. The most commonly used was piperacillin-tazobactam by 41.4%, followed by vancomycin (38.8%), and then carbapenems and aminoglycosides (29.3%) for each. Fluoroquinolones were 16.4%, cephalosporins were 15.5%.

**Table 4:** Empirical antimicrobial therapy used for patients with solid malignancy and BSI

Empirical antibiotic therapy	Frequency	Percent (%)
Initial regimen		
Monotherapy*	33	28.4
Combination therapy*	83	71.6
Aminoglycosides	34	29.3
Cephalosporins	18	15.5
Fluoroquinolones	19	16.4
Carbapenems	34	29.3
Macrolides	2	1.7
Tetracyclines	7	6
Piperacillin-tazobactam	48	41.4
Trimethoprime-ulfamethoxazole	1	0.9
Metronidazole	5	4.3
Vancomycin	45	38.8
Colistin	8	6.9
Amoxicillin-clavulanic acid	1	0.9
Antibiotics other than the mentioned above	7	6
Anti-fungal drugs	16	13.8

*Monotherapy: single antimicrobial, combination: two or more antimicrobials

Isolates of *S. aureus, S. epidermidis, Micrococcus luteus,* and *other Staphylococcal species* showed 100% sensitivity to vancomycin, while *Enterococcus species* was 87.5% sensitive to vancomycin. All *S. aureus* and *S. epidermidis* isolates were fully resistant to benzyl penicillin. Regarding the sensitivity pattern of the sensitivity pattern of *Enterococcus species,* they showed a (63%) sensitivity to ampicillin as in Table 5.

**Table 5:** Antibiotic sensitivity of isolated Gram-positive pathogens from solid malignancy patients during BSI episodes

Gram + bacteria	*Staphylococcus aureus* Sensitivity(UD)	*Staphylococcus epidermis*	*Other staphylococcal species*	*Enterococcus species*	*Micrococcus luteus*
Antibiotics					
Gentamicin	100	82.4	95.7	0 (12.5)	80 (20)
Trimethoprim-Sulfamethoxazole	100	58.8	60.9 (4.3)	UD	40 (60)
Ampicillin	UD	UD	UD	62.5	UD
Amoxicillin / Clavulanic acid	60	5.9	30.4 (4.3)	62.5 (12.5)	100
Clindamycin	80	58.8	39.1	UD	80
Vancomycin	100	100	100	87.5	100
Penicillin – benzyl	0	0	8.7	25	40 (60)
Oxacillin	60	5.9	26.1	12.5	20 (80)
Cefuroxime	60	5.9	30.4 (4.3)	12.5	20 (80)
Erythromycin	60	11.8	26.1	0	40
Tetracycline	100	70.6	73.9 (4.3)	25	40 (60)

*UD Undetermined

The numbers inside the table represent the sensitivity percent for each antibiotic

The number between brackets represents the undetermined percent if present

In the case of *E. coli,* the isolates were fully sensitive to imipenem, meropenem, and amikacin (100℅). They were resistant mainly to ampicillin, in which the sensitivity was approximately 19.5%. However, the sensitivity also decreased with ceftazidime and cefepime in approximately 47.6% and cefotaxime and ceftriaxone in approximately 42.9% in the cultures detected, adding to that the proportion of *E. coli* cultures that tested positive for ESBL is approximately 52.4%. *P. aeruginosa* was sensitive in 83.3% to piperacillin-tazobactam and gentamicin and was highly resistant to imipenem, in which the sensitivity decreased to 50% in detected cases. The *Klebsiella* isolates were 72.2% sensitive to gentamicin, meropenem, and imipenem and had 100% resistance to ampicillin and approximately 44.4% of the *Klebsiella* isolates tested positive for ESBL. *A. baumannii* was 50% sensitive to trimethoprim-sulfamethoxazole and 16.7% sensitive to meropenem and imipenem. Other antibiotics showed less sensitivity, as represented in Table 6. Fungal infections detected in BSI were Candida species that showed a high sensitivity to both caspofungin and flucytosine (83.3%), followed by a sensitivity of 77.8% to voriconazole, as mentioned in Table 7.

**Table 6:** Antibiotic sensitivity of isolated Gram-negative pathogens from solid malignancy patients during BSI episodes

Gram-ve bacteria	*Escherichia coli *Sensitivity(UD)	*Psuedomonas aeruginosa*	*Klebsiella spp.*	*Acinetobacter bumannii*
Antibiotics				
Piperacillin-tazobactam	95.2	83.3	50 (5.6)	16.7
Ceftazidime	47.6	66.7	55.6 (5.6)	16.7
Cefepime	47.6	66.7	55.6 (5.6)	16.7
Imipenem	100	50 (16.7)	72.2	16.7
Meropenem	100	66.7 (16.7)	72.2	16.7
Gentamicin	90.5	83.3	72.2	16.7 (33.3)
Ciprofloxacin	47.6 (4.8)	66.7	55.6 (5.6)	16.7
Trimethoprim-Sulfamethoxazole	19 (47.6)	UD	61.1 (11.1)	50
Amikacin	100	83.3	66.7 (11.1)	UD
Ampicillin	19 (9.5)	UD	0	UD
Amoxicillin/clavulanic acid	66.7 (19)	UD	33.3 (11.1)	0 (66.7)
Cefotaxime	42.9 (4.8)	UD	50 (4.6)	UD
Ceftriaxone	42.9 (4.8)	UD	55.6	0 (66.7)
Levofloxacin	4.8 (90.5)	UD	5.6 (94.4)	0 (50)

*UD* Undetermined

The numbers in the table represent the sensitivity percent for each antibiotic

The number between brackets represents the undetermined percent

**Table 7:** Antibiotic Sensitivity of isolated fungi from solid malignancy patients during BSI episodes

Antifungal/fungi type	Candida species
Fluconazole	55.6 (38.9)
Voriconazole	77.8 (11.1)
Caspofungin	83.3 (16.7)
Flucytosine	83.3 (11.1)

The isolated microorganisms were sensitive to the empiric regimen in 84% of the cases for which there was no need to escalate the regimen.

According to our study, the death rate among the 96 patients was 27.6%. On the contrary, it was 15.5% among patients with multidrug-resistant pathogens. To assess the relationship between some risk factors and death as an outcome of BSI, we used either the chi-square test or Fisher’s exact test. Depending on the resultant *p-value,* we found a significant relationship between shock at presentation and death *(p-value* = 0.010). However, other risk factors such as metastatic tumor, associated comorbidities, and chemotherapy use in the month preceding BSI did not show statistical significance to death (*p-value* > 0.05). Table 8 shows in detail the risk factors and the outcome of patients in their BSI episodes.

**Table 8:** The outcome of patients in relation to certain risk factors

Risk factor	Number of patients survived (%)	Number of dead patients (%)	*p *value
Comorbidities^1^	42 (77.8)	12 (22.2)	0.053*
Cancer with metastasis	28 (70)	12 (30)	0.970*
Recent chemotherapy use^2^	53 (70.7)	22 (29.3)	0.569*
Recent radiotherapy use*	6 (50)	6 (50)	0.067*
Shock at presentation	13 (52)	12 (48)	0.010*
Recent corticosteroid use^2^	10 (58.8)	7 (41.2)	0.175*
Recent use of immunosuppressive medication use^2^	4 (66.7)	2 (33.3)	0.667^f^
Previous antibiotic use^2^	47 (70.1)	20 (29.9)	0.523*
Previous invasive procedures^3^	44 (71)	18 (29)	0.709*

^1^Comorbidities such as diabetes mellitus, hypertension, chronic kidney disease, inflammatory bowel disease, cardiac diseases, thyroid disorders, pulmonary diseases, and neurological disorders

^2^During a month before the occurrence of the blood stream infection

During the ten days preceding the bloodstream infection

*Using the chi-square test

^f^using Fisher’s exact test

The italic *p*-value denotes statistical significance

## Discussion

Most of the studies conducted to search for BSI were conducted in patients with hematologic malignancies and bone marrow transplants; there is a lack of available data on BSI in solid malignancy patients [9, 10]. Therefore, this retrospective study involved solid malignancy patients who experienced one or more BSI during three years to study the epidemiology, etiology, outcome, predisposing factors, and characteristics of each episode of BSI episode in those patients.

BSI episodes were more frequent in patients with breast cancer, accounting for 13.5% compared to other types of malignancies, followed by patients with urinary tract tumors, 10.4% of total cases. A similar study conducted in Spain found that hepatobiliary tumors were the most frequent neoplasm associated with episodes of BSI episodes with 19%, followed by lung tumors (18%). On the contrary, breast cancer scored only 7% [1]. Patients with solid tumors are prone to infections due to obstruction and interference with physical barriers such as skin [11, 12]. In addition, these patients usually have low immunity due to receiving chemotherapy, radiation, and immunosuppressive medications. In addition, they undergo invasive procedures for diagnostic and therapeutic reasons, which in addition to other factors put the patient at increased risk of catching infections with different organisms [13, 14].

A study conducted in Mexico has shown that 14% of patients diagnosed with solid malignancies have developed BSI at least once. Half of those were men and most infections were Gram-negative pathogens. According to the same study, BSIs were more common in GI cancers and were followed by head and neck tumors. Regarding antibiotic susceptibility, gram-negative bacteria develop only 17% resistance to B-lactamase inhibitors, and gram-positive bacteria develop 12% resistance to methicillin resistance in the Latin American population [15].

A study conducted in Australia has shown that patients with solid tumors developed vancomycin resistance to vancomycin among *Enterococci,* infection is observed to be 9%, and resistance among *S. aureus* isolates is 16% [16].

Another study conducted in Greece in 2004 has shown that BSI episodes were associated with healthcare, 35% were nosocomial, 14% were acquired in the community, and 12% of the patients had two or more episodes of drug resistance during hospital admission [9].

A study conducted previously in Spain 2014 has shown that oncology patients admitted to the hospital with inadequate empiric treatment had frequent episodes of multidrug resistance pathogens [1].

A study conducted in Egypt in 2019 focuses on studying resistance to specific antibiotic colistin as a treatment for pneumonia among patients diagnosed with solid malignancy. It shows 8.8% of that developed resistance [17].

Various risk factors are correlated with different clinical pictures, microbial spectrum, and antimicrobial resistance. We identified that approximately 56% of the patients had comorbidities and 64.7% had chemotherapy, and approximately 10% had radiotherapy the month prior to the BSI. Furthermore, we noticed that some patients were on corticosteroids or immunosuppressive therapy or antimicrobials during the month before BSI, as previously reported in the literature [1, 18].

According to our study, we found that 53.4% of the patients had undergone at least one invasive procedure within the ten days prior to their BSI episode, including endoscopy, surgery, catheter or line insertion or removal. Regarding the probable source of the episodes of BSI, the unknown source was the most documented, followed by the catheter-related source predominated by the central line. A previous study conducted in Spain found that cholangitis was the most common source of BSI episodes [1]. A small prospective study involving patients with solid and hematologic malignancies found that catheter-related infections are the most common source of infection [19]. In another study, urinary tract infections were the most frequent source of episodes [9].

According to the causative organism of the episodes of BSI, the results revealed that a Gram-positive bacterium was more frequent in blood cultures than Gram-negative bacteria. Gram-negative bacteria in order of decreasing prevalence were *E. coli, Klebsiella species,* P. *aeruginosa,* and *A. baumannii*. In another study, Gram-negative bacteria were the most documented organisms [2].

*Staphylococcal species*, including coagulase negative *Staphylococcus species*, were on the top of the isolates (33.5%), raising concerns about probable contamination since infection with these organisms is usually skin infection rather than bloodstream infection. In a retrospective study, they found that there was an increase in the incidence of Gram-positive organisms in the last 2 years prior to the study, which was explained by the increase in surgical procedures and the overuse of catheters [9, 20]. Unlike a study conducted in Spain that reported that coagulase-negative *Staphylococci* represented 16% of gram-positive bacteria isolates, they attributed this to the scarce use of prophylactic antibiotics among solid malignancy patients and the decrease in the use of indwelling catheters [1]. Regarding *Candida species*, it was documented in 15.5% of BSI episodes cultured alone or in conjunction with other organisms. Polymicrobial infections accounted for 11.2% of all episodes. A study by the University of New South Wales reported that 22% of the episodes were polymicrobial when isolated [16].

Empirical antimicrobials were defined as the medication prescribed after taking the blood culture and before obtaining the culture result and were chosen according to the patients' risk factors of the patients, clinical manifestations and probable source of BSI.

All patients in this study have received empirical antimicrobials, the majority of them started with combination therapy, b-lactam- b-lactamase inhibitors **(**piperacillin-tazobactam) were more frequently similar to most of the literature, which represented 41.4%, followed by vancomycin, while in previous studies b-lactam- b-lactamase inhibitors accounted 15.5% [1, 4].

In recent years, resistance to antibiotics has increased rapidly, and MDR pathogens have become an issue, especially in immunosuppressed patients [21, 22]. According to our study, MRSA was documented only in two patients. By analyzing the antibiotic sensitivity used on Gram-positive bacteria, the result showed no resistance to vancomycin, similar to many studies conducted in different countries; by studying antimicrobial resistance individually for each bacterium, *Staphylococcus aureus* and *Staphylococcus epidermidis* were completely resistant to benzyl penicillin, and other *Staphylococcus spp.* show low sensitivity to the same antibiotic. A study conducted in Iran showed that *Staphylococcus epidermidis* was susceptible to clindamycin and s*taphylococcus spp.* were 100% clindamycin sensitive, while most of *Staphylococcus aureus* were completely resistant to vancomycin and all Gram-positive isolates showed sensitivity to cloxacillin [2].

Regarding the sensitivity and resistance profile of Gram-negative bacteria to antibiotics, resistance of *E. coli* to fluoroquinolones is a problem in many countries [2]. About 47.6% of the *E. coli* isolates in our study were sensitive to ciprofloxacin. In our study, *E*.*coli* isolates were sensitive to cephalosporins in a percentage between 42–47%. All *E. coli* were sensitive to imipenem and meropenem in this study. According to the literature, studies conducted between 2006 and 2013 show that approximately 95% of *E. coli* were sensitive to carbapenems [23]. However, this percentage has started to decrease in recent years, as in a study that showed sensitivity to carbapenems in a percentage of more than 65%, as in one of the recent studies [2]. The *Klebsiella species* were sensitive to cephalosporins in a percentage ranging from 50–55% and approximately 72% sensitivity to carbapenems in this study, which is suggested to be caused by the complicated course of the patients due to recurrent admissions to the ICU and an increase in the length of hospitalization [24]. A study conducted in Egypt showed that the sensitivity of *Klebsiella species* to carbapenems was approximately 80% [25]. A study conducted showed 90–100% sensitivity to cephalosporins and carbapenems, inconsistent with other studies [2, 23]. *P. aeruginosa* showed a sensitivity of approximately 67% for ciprofloxacin and ceftazidime and approximately 50% for imipenem and 67% for meropenem, similar to a Saudi Arabian study that showed that sensitivity to imipenem was approximately 52.4% [26].

## Strengths and limitations

Suppose that we want to talk about the strengths of this study. In that case, we mention that it is the first study in the West Bank, Palestine, to discuss the microbial spectrum and sensitivity profile in solid malignancy patients. We also mention the importance of antibiotic stewardship, especially with more resistant pathogens, which is considered a global problem.

As our study was a retrospective study, it was difficult to find fully complete patient data. In addition to that, we studied a very specific concept in patients with solid malignancy, which made it challenging to find other related data. In addition to that, approximately 17% of the sample was excluded due to missing data from the hospital system or loss of follow-up for some patients.

## Conclusions

The findings of this investigation confirm the prevalent BSI seen in patients with solid malignancies and demonstrate a significant percentage of antibiotic resistance. As a result, patients will face different infections due to multiple hospital admissions to perform different diagnostic and therapeutic procedures, increasing the risk of contamination. Patients who experience bloodstream infection may have poor outcomes, especially when dealing with microorganisms that resist multiantimicrobials. Many of the infections were related to catheters; Blood cultures before and after any procedure should be considered and followed according to the result and decrease the use of invasive procedures as much as possible.

Finally, stewardship programs that focus on the utility of antimicrobials and aim to reduce the abusive use of antibiotics should be implemented in hospitals, especially in oncology departments.

## Supplementary Information

**Additional file 1. **Data collection form. This is the final version of the English version that was used to obtain data that will help to to assess the microbial spectrum and antimicrobial sensitivity and the overall outcome related to many clinical risk factors in patients with solid tumor patients seeking care in a referral hospital as an experience from a developing country.

## Data Availability

The data from our surveillance are not available on the public domain due to privacy and ethical restrictions, but anyone interested in using the data for scientific purposes is free to request permission from the corresponding author. Dr. Sa’ed H. Zyoud (saedzyoud@yahoo.com). This manuscript forms part of a Doctor of Medicine graduation project submitted to An-Najah National University and the abstract was published as part of self-archiving in institutional repositories (that is, university repository: https://repository.najah.edu/handle/20.500.11888/16054).

## Abbreviations

BSI: Bloodstream infection
Spp.: Species
MDR: Multidrug resistance
ICU: Intensive care unit
IRB: Institutional Review Boards
