# Supplementary material for: Microbial spectrum and drug resistance profile in solid malignancies in a large tertiary hospital from Palestine
Source: BMC Infect Dis. 2022 Apr 18;22:385. doi: 10.1186/s12879-022-07375-6 (PMC9014612; doi:10.1186/s12879-022-07375-6)
Supplement: Supplementary file 1 — Additional file 1. Data collection form. This is the final version of the English version that was used to obtain data that will help to to assess the microbial spectrum and antimicrobial sensitivity and the overall outcome related to many clinical risk factors in patients with solid tumor patients seeking care in a referral hospital as an experience from a developing country. [file 12879_2022_7375_MOESM1_ESM.docx]

**Additional file 1.** **Data collection form. This is the final version of the** **English version that was used to obtain data which will help to to assess the microbial spectrum and antimicrobial sensitivity and the overall outcome related to many clinical risk factors in patients with solid tumor patients seeking care in a referral hospital as an experience from a developing country.**

**Patient’s demographic features**

| **Patient’s age** |  |
| --- | --- |
| **Gender** |  |
| **Underlying solid tumor** |  |

**Type of underlying solid tumor in patients with bloodstream infections BSIs: {Types of cancer in this table are only examples}**

| **Type of underlying tumor** | **No. of patients** | **Number of BSIs episodes** | **Isolated pathogen** | **Source of infection** |
| --- | --- | --- | --- | --- |
| Gastric cancer |  |  |  |  |
| Colon |  |  |  |  |
| hepatobiliary |  |  |  |  |
| Breast |  |  |  |  |
| others |  |  |  |  |

**Factors associated with BSIs. (Multiple factors may coexist in the same patients)**

| **Risk factors** | **Episodes*** | **Percentage of risk factor among all patients** |
| --- | --- | --- |
| Recent chemotherapy * |  |  |
| Recent Radiotherapy * |  |  |
| Previous invasive procedure |  |  |
| Previous antibiotic therapy |  |  |
| Recent medications |  |  |

**Definitions:**

**Episode*:** used for each separate event of BSIs, more than 4 weeks between each episode.

**Recent chemotherapy*:** over the preceding 30 days.

**Recent radiotherapy*:** over the preceding 30 days.

**Previous invasive procedure*:** performed in the preceding 10 days,

Some examples: Peripheral or central venous catheter, urinary catheter, bronchoscope and colonoscopy.

**Recent medications*:** using corticosteroids,immunosuppressive or hormonal therapy in the previous 30 days of having positive culture.

**Recent antibiotic use*:** over the preceding 2 days before having suspected BSIs.

**Types of organisms isolated and its associated mortality:**

| **Pathogen** | **Number of BSIs** | **Number of deaths** |
| --- | --- | --- |
| Gram positive-Bacteria |  |  |
| Gram negative-Bacteria |  |  |
| Fungi |  |  |
| Polymicrobial |  |  |

**Clinical factors associated with mortality**

| **Clinical factors** | **Number of episodes** | **Mortality** |
| --- | --- | --- |
| Presence or absence of neutropenic fever |  |  |
| Initial empirical antibiotic regimen |  |  |
| Presence /absence of shock |  |  |
